# *Loxostigma puhoatense* (Gesneriaceae), a new species from North Central Vietnam

**DOI:** 10.3897/phytokeys.151.49473

**Published:** 2020-06-12

**Authors:** Ngọc-Sâm Lý, Danh-Hùng Nguyễn, Thị-Hương Lê, Minh-Hợi Trần, Ngọc-Đài Đỗ, Bá-Vương Trương, Stephen Maciejewski

**Affiliations:** 1 Institute of Tropical Biology, Vietnam Academy of Science and Technology (VAST), 85 Tran Quoc Toan, District 3, Ho Chi Minh City, Vietnam Graduate University of Science and Technology Hanoi Vietnam; 2 Graduate University of Science and Technology, VAST, 18 Hoang Quoc Viet, Cau Giay District, Hanoi, Vietnam Institute of Tropical Biology, Vietnam Academy of Science and Technology Ho Chi Minh Vietnam; 3 School of Natural Science Education, Vinh University, 182 Le Duan, Vinh City, Nghe An Province, Vietnam Vinh University Vinh Vietnam; 4 Institute of Ecology and Biological Resources, VAST, 18 Hoang Quoc Viet, Cau Giay District, Hanoi, Vietnam Institute of Ecology and Biological Resources Hanoi Vietnam; 5 Nghe An College of Economics, 51 Ly Tu Trong, Vinh City, Nghe An Province, Vietnam Nghe An College of Economics Vinh Vietnam; 6 The Gesneriad Society, Inc., 1122 East Pike Street, PMB 637, Seattle, Washington, USA The Gesneriad Society Seattle United States of America

**Keywords:** *
Briggsia
*, *
Didymocarpus
*, Gesneriaceae, *
Loxostigma
*, taxonomy, Vietnam

## Abstract

*Loxostigma puhoatense* N.D. Do et al., **sp. nov.**, a new species of Gesneriaceae, is described and illustrated from Pu Hoat Nature Reserve, Nghe An Province, Vietnam. This species is morphologically similar to *L. dongxingensis* and *L. damingshanensis* in the plant habit, indumentum system of vegetative and reproductive characters, shape of leaf blades, calyx, pistil but differs from the latter two by the abaxially reddish-purple leaf blade with pubescent along veins, lateral veins 11–19, shorter white to pale yellowish-white corolla (3.7–3.8 cm long) with purple-spotted and glabrous inside, longer abaxial stamens in 2–2.1 cm, shorter and densely glandular-puberulent ovary in 1–1.2 cm, and sparsely glandular-puberulent style. Data on distribution, ecology, phenology, and vernacular of the new species are provided.

## Introduction

The genus *Loxostigma* C.B. Clarke is in the family Gesneriaceae, with at least 11 species recognized including one species from *Didymocarpus* Wallich and three species of the former *Briggsia* Craib (Möller et al. 2014) based on molecular and morphological evidences (Pan 1988; Wu et al. 2012; Möller et al.2011; Möller et al. 2014). It is distributed mainly in Southern and Southeastern Asia, and most of the species have restricted distributions in southwestern China (Wang 1983; Wei et al. 2010; Möller et al. 2014, 2016). There are four currently known species of the genus in Vietnam, namely *Loxostigma dongxingensis* (Chun ex K.Y.Pan) Mich. Möller & Y.M.Shui, *L. fimbrisepalum* K.Y.Pan, *L. glabrifolium* D.Fang & K.Y.Pan and *L. griffithii* (Wight) C.B. Clarke (Pham 2000; Vu 2005; Do and Vu 2011; Möller et al. 2014; Do et al. 2016).

During our recent botanical surveys of Pu Hoat Nature Reserve (NR), Nghe An, Vietnam, an unknown species of *Loxostigma* with the seeds having 1-hairlike appendage at each end was collected by the authors in 2018–2019. We conducted a critical examination of the specimens, and made a comparison with type material and protologues of presumed closely related species in Vietnam and neighboring countries (e.g. Wang 1983; Wang and Pan 1982; Pan 1988; Wang et al. 1998; Grierson and Long 2001; Li and Wang 2004; Wei et al. 2010; Sinha and Datta 2016). We discovered that these specimens were different from the other known *Loxostigma* species and presented the unknown taxon which shows similarities with *L. dongxingensis* and *L. damingshanensis* (L.Wu & B.Pan) Mich.Möller & H.Atkins in the same plant habit, indumentum of stem and shape of flower. However, it shows significant differences in its vegetative and floral structures (see Table 1) and is described here as a new species to science.

## Material and methods

All measurements and description of the new species are based on living flowering material and herbarium specimens collected from the type locality, supplemented with type material from the following herbaria: HITBC, HN, IBK, IBSC, K, KUN, P, PE, VNM and VNMN (herbarium codes follow Thiers (2018)) as well as digitized specimen images of *Loxostigma* species available on the web from Muséum National d’Histoire Naturelle (https://science.mnhn.fr/), Chinese Virtual Herbarium (http://www.cvh.ac.cn/) and Jstor Global Plant (https://plants.jstor.org/). All morphological characters were studied under dissecting microscopes and are described using the terminology presented by Wang et al. (1998), Wei et al. (2010) and Beentje (2016).

## Taxonomic treatment

### Loxostigma puhoatense

Taxon classificationPlantaeLamialesGesneriaceae

N.D. Do, N.S. Ly, D.H. Nguyen & T.H. Le sp. nov.

DAFDFBBA-B567-5C70-B27D-B34E48D86154

urn:lsid:ipni.org:names:77209909-1

Figs 1 , 2

#### Diagnosis.

This species is most similar to *L. dongxingensis* and *L. damingshanensis* in the plant habit, indumentum system of stem, leaves, shape of leaf blades, calyx and pistil but differs from the latter two in the leaf blade with abaxially reddish-purple and number of lateral veins, longer peduncle, shorter white to pale yellowish-white corolla with purple-spotted inside, longer abaxially stamens that are lower adnate above corolla tube base, shorter and glandular-puberulent ovary, and sparsely glandular-puberulent style.

**Figure 1.**
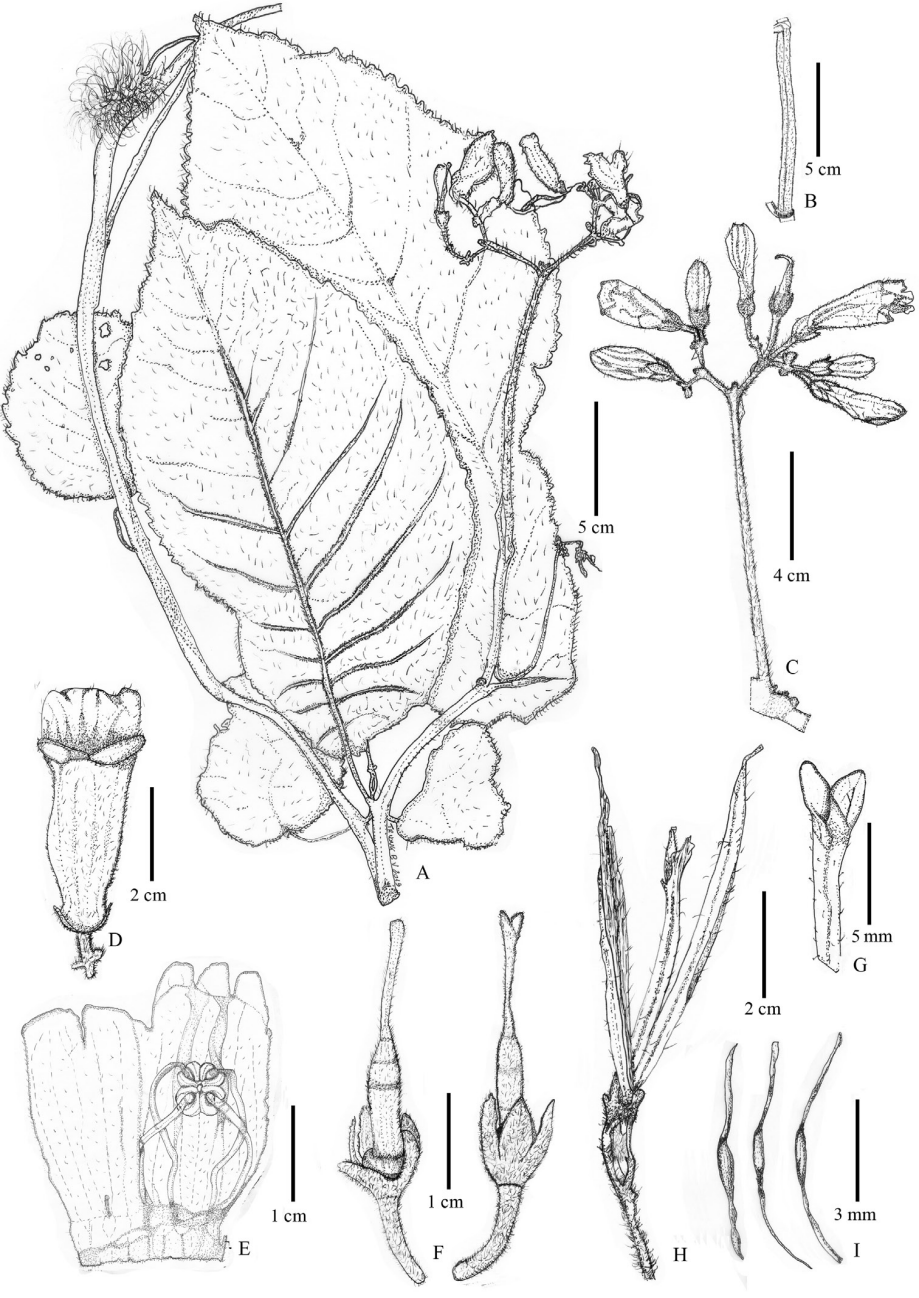
*Loxostigma puhoatense* (from the holotype). **A** mature plant with flowers **B** a part of stem **C** inflorescence **D** dorsal view of flower **E** opened flower showing stamens **F** gynoeciums **G** detail of stigma (from dry specimen) **H** infructescence **I** seeds. Drawn by Ba-Vuong Truong from Do Ngoc Dai, Nguyen Danh Hung, Le Thi Huong, DHH 1021.

#### Type:

Vietnam. Nghe An Province: Tien Phong Commune, Na Chang Village, Pu Hoat NP, 19°46'06"N, 109°55'08"E, atl. 781 m, 04 October 2018, *Do Ngoc Dai, Nguyen Danh Hung, Le Thi Huong*, *DHH 1021* (holotype: VNM, isotype: P).

#### Description.

Epiphyte, evergreen, perennial herbs with rhizomes. Rhizome internodes up to 20 cm long, 3.5–4 mm in diam. ***Stem*** simple, borne from a node of the rhizome, shallow angular at the middle internodes, terete toward both ends, 28.5–55 cm long, 3.5–7 mm in diam., pale green, somewhat pubescent. ***Leaves*** opposite, spread along stem, unequal in a pair; petiole cylindrical, adaxially sunken, greenish to purplish, abaxially rounded and greenish, 1–3.3 cm long, 3–6 mm in diam., pubescent; leaf blade broadly elliptic to elliptic-ovate, chartaceous when dried, 4.2–25.5 × 3–15.5 cm [11.3–25.5 × 8.9–15.5 cm in larger leaves, 4.2–9.3 × 3–6.2 cm in smaller leave], adaxially light green, abaxially reddish purple, sparsely pubescent on both surface, pubescent on the midrib and lateral veins of the abaxial surface, lateral veins 11–19 pairs, base usually oblique, subcordate, margin serrate to indistinctly denticulate, apex acuminate. ***Cymes*** axillary, subterminal, 2–3-branched, 3–25-flowered; peduncle 9.2–17.5 cm long, 2–3 mm in diam., pale greenish, densely pubescent and glandular-pubescent. ***Bracts*** ovate to oblong-ovate, pale greenish-white tinted, 5–8.5 × 2.8–3.5 mm, adaxially glabrous, abaxially densely pubescent and glandular-pubescent, margin somewhat denticulate. ***Pedicel*** 1.1–1.4 cm long, ca. 1.5 mm in diam., greenish-white, densely pubescent and glandular-pubescent. ***Calyx*** 5-sect from the base, segments equal, whitish, narrowly ovate, 6–7 × 2–2.5 mm, adaxially glabrous, abaxially densely pubescent and glandular-pubescent, margin entire, apex acute. ***Corolla*** somewhat campanulate, white to pale yellowish-white, gibbous abaxially, with purple spots inside, 3.7–3.8 cm long, outside densely glandular-pubescent, inside glabrous; ***corolla tube*** 2.7–2.9 × 1.4–1.6 cm; ***corolla limb*** distinctly 2-lipped, adaxial lip 5–6 mm long, 2-lobed, lobes semi-orbicular, 5–5.5 × 7–7.5 mm, deflexed, apex rounded; abaxial lip3-lobed, 10–11 mm long, lobes semi-orbicular, 4–5.5 × 4–5 mm, deflexed, apex rounded. ***Stamens*** 4, adaxial stamens adnate to 7–8 mm above corolla tube base, 15–16 mm long, abaxial ones adnate to 6–7 mm above corolla tube base, 20–21 mm long; ***filaments*** linear, white, glabrous, slightly curved; ***anthers*** sub-globose, theca coherent apically in pairs, pale cream; ***staminode*** one, adnate to 7–8 mm above corolla tube base, ca. 2 mm long. ***Disc*** ring-like, subentire, ca. 2.5mm high. ***Pistil*** 2.2–2.4 cm long; ***ovary*** oblong, greenish, 10–12 × 1.8–2 mm, densely glandular-puberulent; ***style*** linear, pale greenish, 9–10 × ca. 1 mm, sparsely glandular-puberulent; ***stigma*** 2, equal, 2-lipped, undivided. ***Capsule*** 6–6.5 cm long, 2–3mm in diam., oblong-linear, straight, not twisted, glabrous, blackish-brown, dehiscing loculicidally to base, valves 2. ***Seeds*** linear, 2–2.5 mm long, brown, with appendages on both ends of the seeds; ***appendages*** 2.5–3 mm long.

#### Distribution and habitat.

**Figure 2.**
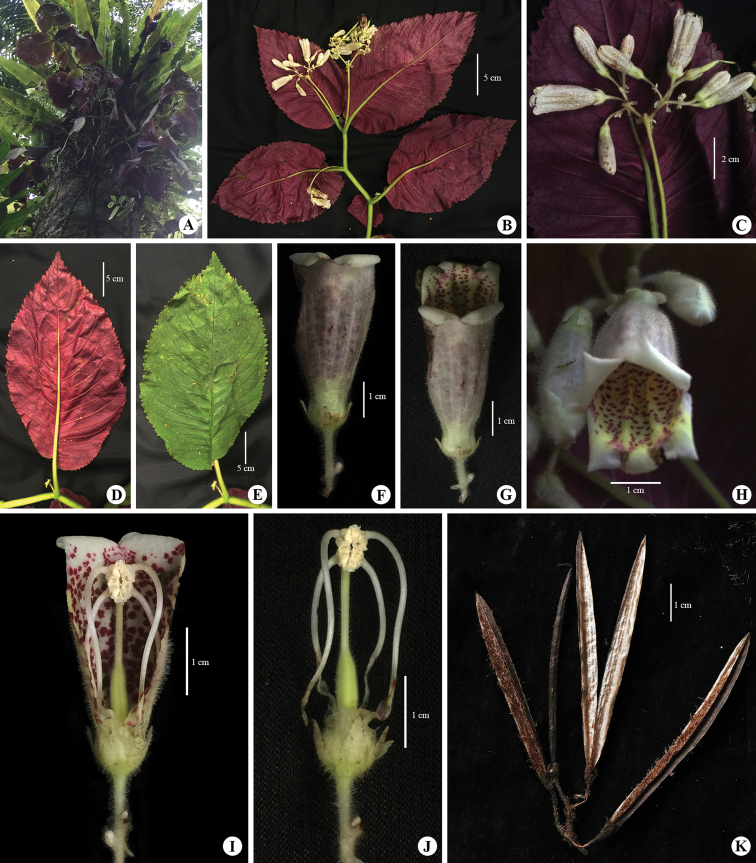
*Loxostigma puhoatense*. **A** plant in natural habitat **B** apical part of flowering plant showing inflorescences (with flowers and young fruits) and leaves **C** inflorescence **D** abaxial leaf **E** adaxial leaf **F** flower (from below) **G** flower (top view) **H** flower (front view) **I** longitudinal section with upper lip removed **J** stamens with gynoecium and calyxes **K** dried fruits. Photos by Ngoc-Dai Do, plate by Ngoc-Sam Ly.

*Loxostigma puhoatense* is currently known from a single population with eight mature plants, in tropical evergreen broad-leaf forests, Pu Hoat Nature Reserve, Nghe An Province. More data is needed to determine conservation status. It is an epiphytic plant (Fig. 2A), growing on the surface of *Ficus* sp. (Moracaeae), and is associated with fern (e.g. *Asplenium nidus* L. (Aspleniaceae), epiphytic herbs (e.g. *Aeschynanthus acuminatus* Wall. ex A. DC. (Gesneriaceae), *Pothos chinensis* (Raf.) Merr. (Araceae) and is dominated by *Beilschmiedia ferruginea* H.Liu, *Cinnamomum polyadelphum* (Lour.) Kosterm., *C. tamala* (Buch.-Ham.) T.Nees & Eberm., *Lithocarpus balansae* (Drake) A. Camus, *Syzygium grande* (Wight) Walp., *S. odoratum* (Lour.) DC., *Gordonia axillaris* (Roxb. ex Ker Gawl.) Endl…

#### Phenology.

Flowering in October–December and fruiting November–February.

#### Etymology.

The specific epithet “*puhoatense*” is derived from the type locality.

#### Other specimens examined (Paratypes).

Vietnam. Nghe An Province: Pu Hoat NP, 24 February 2019, 19°46'06"N, 109°55'07"E, *Do Ngoc Dai, Nguyen Danh Hung, Nguyen Thi Huong*, DHH 1162 (VNM).

#### Vernacular name.

Vietnamese language: Xuyến thư pù hoạt

#### Taxonomic notes.

*Loxostigma puhoatense* is morphologically similar to *L. dongxingensis* and *L. damingshanensis* in the plant habit, somewhat pubescent stem, elliptic to ovate leaf lades, narrowly ovate calyx with margin entire, the same shape of flower. However, the new species differs from both of them in the abaxially reddish-purple leaf blade with pubescent along veins (vs green, villous of *L. dongxingensis* and pilose of *L. damingshanensis*), lateral veins 11–19 pairs (vs. 6–10 pairs and 5–10 pairs of the latter two, respectively), shorter white to pale yellowish-white corolla (3.7–3.8 cm long) with purple-spotted and glabrous inside (vs. 4–5 cm long, yellow, inside spotted or not and sparsely pubescent only on lobes of adaxial lip of *L. dongxingensis*, and 4.8–6 cm long, yellow, inside purple stripes and sparsely pubescent only on lobes of abaxial lip, of *L. damingshanensis*), longer abaxial stamens in 2–2.1 cm long (vs. ca. 18 cm and ca. 14 cm in the latter two, respectively), shorter and densely glandular-puberulent ovary in 1–1.2 cm long (vs. ca. 2 cm, glabrous of *L. dongxingensis* and ca.1.8 cm, densely glandular-pubescent of *L. damingshanensis*), and sparsely glandular-puberulent style (vs. glandular-pubescent of *L. dongxingensis* and *L. damingshanensis*). The staminode of *L. puhoatense* is ca. 2 mm long (vs. ca. 1 mm of *L. dongxingensis* and ca. 1.5 mm of *L. damingshanensis*) and adnate above corolla tube base in 0.7–0.8 mm long (vs. ca. 1.3 cm of *L. dongxingensis* and ca. 1.8 cm of *L. damingshanensis*). Furthermore, the densely pilose petiole of *L. dongxingensis* is distinguished with the somewhat pubescent petiole of *L. puhoatense* and *L. damingshanensis*. The unbranched cyme and inconspicuous or absent of bract of *L. damingshanensis* are distinct with the branched cyme and 2- somewhat ovate bracts of the remained two. A detailed morphological comparison between *L. puhoatense*, *L. dongxingensis*, and *L. damingshanensis* is provided in Table 1.

**Table 1.** Morphological comparison of *Loxostigma puhoatense*, *L. dongxingensis* and *L. damingshanensis*.

Characters	*L. puhoatense*	*L. dongxingensis*	*L. damingshanensis*
Stem	somewhat pubescent	densely pubescent	sparsely pubescent
Leaf blade	broadly elliptic to elliptic-ovate, abaxially reddish-purple, sparsely pubescent on both surfaces, pubescent along veins, lateral vein 11–19 pairs	elliptic to ovate or obovate, abaxially green, adaxially appressed puberulent to pubescent, abaxially pubescent, villous along veins, lateral vein 6–10 pairs	elliptic-ovate, abaxially green, sparsely pubescent on both surfaces, pilose along veins, lateral veins 5–10 pairs
Petiole	pubescent	densely pilose	sparsely pubescent
Cyme	branched	branched	unbranched
Peduncle	9.2–17.5 cm long, densely pubescent and glandular-pubescent	4–10 cm long, pubescent	4–8 cm long, sparsely glandular-pubescent
Bract	ovate to oblong-ovate, densely pubescent and glandular-pubescent, margin somewhat denticulate	narrowly ovate to narrowly oblong, pubescent, margin entire	inconspicuous or absent
Pedicel	densely glandular-pubescent	pubescent	sparsely glandular-pubescent
Calyx	outside densely glandular-pubescent	outside pubescent to villous	outside densely glandular-pubescent
Corolla	white to pale yellowish-white with purple-spotted inside, 3.7–3.8 cm long, outside densely glandular pubescent, inside glabrous	yellow, inside spotted or not, 4–5 cm long, outside sparsely pubescent, inside sparsely pubescent only on lobes of adaxial lip	yellow, inside with purple stripes, 4.8–6 cm long, outside sparsely glandular-pubescent, inside sparsely pubescent only on lobes of abaxial lip
Staminode	ca. 2 mm long, adnate 7–8 mm above corolla tube base	ca. 1 mm long, adnate to ca. 13 mm above corolla tube base	ca. 1.5 mm long, adnate to ca. 18 mm above corolla tube base
Abaxial stamens	2–2.1 cm long	ca. 1.8 cm long	ca. 1.4 cm long
Ovary	1–1.2 cm long, densely glandular-puberulent	ca. 2 cm long, glabrous	ca. 1.8 cm long, densely glandular-pubescent
Style	sparsely glandular-puberulent	glandular pubescent	glandular-pubescent

### A key to known species of *Loxostigma* occurring in Vietnam

**Table:** 

1	Calyx lobes narrowly ovate. Peduncle somewhat pubescent. Stem usually less than 60 cm tall	**2**
–	Calyx lobes ovate to broadly ovate or oblanceolate. Peduncle somewhat puberulent. Stem usually ≥ 60 cm tall	**4**
2	Stem pubescent. Leaf blades somewhat hairs on both surfaces, abaxially pubescent or villous along veins, margin denticulate to serrate. Corolla white or yellow. Staminode > 1 mm long. Abaxial stamens > 1.5 cm long. Ovary > 10 mm long	**3**
–	Stem sparsely puberulent or glabrescent. Leaf blades glabrous, abaxially sparsely puberulent along veins, margin denticulate to entire. Peduncle sparsely puberulent. Corolla white to yellow. Staminode ca. 0.5 mm long. Abaxial stamens ca. 1 cm long. Ovary 7–8 mm long.	***L. glabrifolium***
3	Leaf blades abaxially reddish-purple, sparsely pubescent on both surfaces; lateral veins 11–19 pairs; petiole pubescent. Margin of bract denticulate. Corolla white to pale yellowish-white with purple-spotted inside, 3.7–3.8 cm long. Abaxial stamens 2–2.1 cm long. Staminode ca. 2 mm long. Ovary 1–1.2 cm long, densely glandular-puperulent	***L. puhoatense***
–	Leaf blades green; adaxially appressed puberulent to pubescent, abaxially pubescent, villous along veins; lateral vein 6–10 pairs; petiole densely pilose. Margin of bract entire. Corolla yellow, inside spotted or not, 4–5 cm long. Abaxial stamens ca. 1.8 cm long. Staminode ca. 1 mm long. Ovary ca. 2 cm long, glabrous	***L. dongxingensis***
4	Leaves ovate to broadly elliptic, margin repand to serrulate. Margin of bract denticulate to repand. Calyx ovate. Corolla white-lilac, inside purple spots and glabrous	***L. fimbrisepalum***
–	Leaves elliptic to ovate or obovate, margin serrate to crenate-serrulate. Bract with irregularly dentate margin. Calyx broadly ovate or oblanceolate. Corolla yellowish, inside purplish to brownish spots and puberulent	***L. griffithii***

## Supplementary Material

XML Treatment for Loxostigma puhoatense

## References

[B1] Beentje H (2016) The Kew Plant Glossary, an illustrated dictionary of plant terms (2nd edition). Royal Botanic Gardens, Kew.

[B2] Do TX Vu XP (2011) A new record of species *Loxostigma fimbrisepalum* K.Y. Pan (Gesneriaceae Dumort) for the flora of Vietnam. Tap Chi Sinh Hoc 33: 45–47. [In Vietnamese with English summary] 10.15625/0866-7160/v33n4.779

[B3] Do VT Li S Wei YG Fu LF Wen F (2016) New records and keys to species of *Hemiboea* and *Loxostigma* (Gesneriaceae) for the flora of Vietnam. Taiwania 61(4): 369–374.

[B4] Grierson AJC Long DG (2001) Flora of Bhutan 2: 1–1675. Royal Botanic Gardens, Edinburgh.

[B5] Li ZY Wang YZ (2004) Plants of Gesneriaceae in China. Henan Science & Technology Publishing House, Zhengzhou. [In Chinese]

[B6] Möller M Forrest A Wei YG Webber A (2011) A molecular phylogenetic assessment of the advanced Asiatic and Malesian didymocarpoid Gesneriaceae with focus on non-monophyletic and monotypic genera. Plant Systematics and Evolution 292(3-4): 223–248. 10.1007/s00606-010-0413-z

[B7] Möller M Chen WH Shui YM Atkins H Middleton DJ (2014) A new genus of Gesneriaceae in China and the transfer of *Briggsia* species to other genera. Gardens’ Bulletin (Singapore) 66: 195–205.

[B8] Möller M Wei YG Wen F Clark JL Weber A (2016) You win some you lose some: Updated generic delineations and classification of Gesneriaceae – implications for the family in China. Guihaia 36: 44–60.

[B9] Pan KY (1988) New taxa of *Briggsia* Craib (Gesneriaceae) from China. Zhiwu Fenlei Xuebao 26: 450–457. [In Chinese]

[B10] Pham HH (2000) Gesneriaceae. In: Pham HH (Ed.) An Illustrated Flora of Vietnam,Vol.3. Young Publishing House, Tp. Ho Chi Minh, 2–29.[In Vietnamese with English summary]

[B11] Sinha BK Datta S (2016) Taxonomic account on the family Gesneriaceae in Northeast India. Nelumbo 58(0): 1–43. 10.20324/nelumbo/v58/2016/105932

[B12] Thiers B (2018) (continuously updated) Index Herbariorum: A global directory of public herbaria and associated staff. New York Botanical Garden’s Virtual Herbarium. http://sweetgum.nybg.org/science/ih/ [accessed 20 February 2019]

[B13] Vu PX (2005) Gesneriaceae. In: Nguyen BT (Ed.) Checklist of plant species of Vietnam, Vol.3. Agriculture Publishing House, Hanoi, 235–246. [In Vietnamese]

[B14] Wang WT (1983) Genus Novum Gesneriacearum E Guangxi. Guihaia 3(1): 1–6.

[B15] Wang WT Pan KY (1982) Notulae de Gesneriaceis Sinensibus III. Bulletin of Botanical Research 2(2): 121–152.

[B16] Wang WT Pan KY Li ZY (1998) Gesneriaceae. In: Wu ZY Raven PH (Eds) Flora of China, Vol.18. Science Press, Beijing, and Missouri Botanical Garden Press, St. Louis, 276–280, 390–393.

[B17] Wei YG Wen F Möller M Monro A Zhang Q Gao Q Mou HF Zhong SH Cui C (2010) Gesneriaceace of South China. Guangxi Institute of Botany, Guangxi Zhuang Autonomous Region and the Chinese Academy of Sciences, Guangxi Science and Technology Press, Nanning, Guangxi, China.

[B18] Wu L Pan B Yang JC Xu WB (2012) *Briggsia damingshanensis* (Gesneriaceae), a new species from Guangxi, China. Annales Botanici Fennici 49(1–2): 79–82. 10.5735/085.049.0111

